# CRISPR/Cas9 Ribonucleoprotein-Based Genome Editing Methodology in the Marine Protozoan Parasite *Perkinsus marinus*

**DOI:** 10.3389/fbioe.2021.623278

**Published:** 2021-04-09

**Authors:** Raghavendra Yadavalli, Kousuke Umeda, Hannah A. Waugh, Adrienne N. Tracy, Asha V. Sidhu, Derek E. Hernández, José A. Fernández Robledo

**Affiliations:** ^1^Bigelow Laboratory for Ocean Sciences, East Boothbay, ME, United States; ^2^National Research Center for Protozoan Diseases, Obihiro University of Agriculture and Veterinary Medicine, Obihiro, Japan; ^3^Southern Maine Community College, South Portland, ME, United States; ^4^Colby College, Waterville, ME, United States

**Keywords:** *Perkinsus marinus*, oral adjuvant, heterologous expression system, CRISPR/Cas9, protozoan, transfection, oral vaccines

## Abstract

*Perkinsus marinus* (Perkinsozoa), a close relative of apicomplexans, is an osmotrophic facultative intracellular marine protozoan parasite responsible for “Dermo” disease in oysters and clams. Although there is no clinical evidence of this parasite infecting humans, HLA-DR4^0^ transgenic mice studies strongly suggest the parasite as a natural adjuvant in oral vaccines. *P. marinus* is being developed as a heterologous gene expression platform for pathogens of medical and veterinary relevance and a novel platform for delivering vaccines. We previously reported the transient expression of two rodent malaria genes *Plasmodium berghei HAP2* and *MSP8*. In this study, we optimized the original electroporation-based protocol to establish a stable heterologous expression method. Using 20 μg of *p*PmMOE[MOE1]:GFP and 25.0 × 10^6^ *P. marinus* cells resulted in 98% GFP-positive cells. Furthermore, using the optimized protocol, we report for the first time the successful knock-in of GFP at the C-terminus of the *PmMOE1* using ribonucleoprotein (RNP)-based CRISPR/Cas9 gene editing methodology. The GFP was expressed 18 h post-transfection, and expression was observed for 8 months post-transfection, making it a robust and stable knock-in system.

## Introduction

*Perkinsus marinus* (original name *Dermocystidium marinum*), first described in 1950 as infecting the eastern oyster (*Crassostrea virginica*), is still a constant threat to the oyster industry (Mackin et al., 1950; Andrews, 1996; Perkins, 1996). In North America, *P. marinus* and *Perkinsus chesapeaki* can coexist in the same bivalve host (McLaughlin and Faisal, 1998; Coss et al., 2001a, b; Pecher et al., 2008; Reece et al., 2008; Arzul et al., 2012). In the oysters, the parasite is taken up by hemocytes and uses them as a vehicle for migration into other host tissues (Lau et al., 2018a; Schott et al., 2019; Yadavalli et al., 2020). Studies based on intracellular structures and phylogeny suggest *P. marinus* as a close relative to the apicomplexan, a lineage leading to intracellular parasitism having shared genomic and physiological affinities (Matsuzaki et al., 2008; Joseph et al., 2010; Bachvaroff et al., 2011; Fernández Robledo et al., 2011; Van Voorhis et al., 2016).

Human exposure to *Perkinsus* spp. by consuming infected oysters/clams is likely to occur based on the high prevalence of the parasite in oysters (Marquis et al., 2015, 2020). Nevertheless, to our knowledge, the effect of consumption of *P. marinus*-infected oysters has not been investigated in humans. Interestingly, in studies using humanized mice expressing HLA-DR4^0^ genes and lacking expression of mouse MHC-class II genes (DR4.EA^0^), we reported that DR4.EA^0^ mice did not develop any detectable pathology or systemic inflammation (Wijayalath et al., 2014). Notably, naïve (unfed) DR4.EA^0^ mice had antibodies (IgM and IgG) reacting against *P. marinus*, whereas parasite-specific T-cell responses were undetectable. Upon oral feeding with *P. marinus*, parasite-specific IgM and IgG antibodies were boosted with parasite-specific cellular (INFγ) responses detected in the spleen, suggesting *P. marinus* as a natural adjuvant (Wijayalath et al., 2014).

Our group is focused on developing molecular tools to establish *P. marinus* as a heterologous expression system to express genes of pathogens of medical and veterinary relevance. Previously, we built the plasmid *p*MOE[MOE1]:GFP (formerly known as *p*MOE:GFP) by expanding 1 kb each of 5′ and 3′ flanking regions for *PmMOE1* coding sequence tagged with *GFP*, developing an electroporation-based transfection protocol to deliver the plasmid, and successfully showing a single integration event into the genome via non-homologous recombination (Fernández Robledo et al., 2008). Recent studies using developed electroporation-based transfection protocol and *p*MOE[MOE1]:GFP plasmid reported possibilities of plasmid fragmentation and transposable element-dependent genome integration (Faktorová et al., 2020). Considering the phylogenetic relationship of *P. marinus* with apicomplexans, we transfected *P. marinus* with plasmids carrying *Plasmodium berghei HAP2* and *MSP8* and observed transient expression of both genes (Cold et al., 2016). However, we fell short of replicating 37.8% efficiency when the transfection methodology was developed (Fernández Robledo et al., 2008).

To our knowledge, other than the transfection using *p*MOE[MOE1]:GFP-derived plasmids, currently, there are no systems for functional studies of *P. marinus* genes like gene knock-out. The clustered regularly interspaced short palindromic repeats (CRISPR) and CRISPR-associated protein 9 (Cas9) system is a powerful tool for editing genomes (Jinek et al., 2012; Lander, 2016). CRISPR/Cas9 technology utilizes machinery such as Cas9 protein, an RNA-guided endonuclease protein, as well as a guide RNA (gRNA) for the nuclease to generate a double-strand break, which is repaired by nonhomologous end joining (NHEJ) and random mutations incorporated to disrupt the target gene (Mali et al., 2013; Bortesi and Fischer, 2015). However, to knock-in a gene of interest, a donor DNA (dDNA) molecule with homologous templates on either side of the knock-in sequence is required in addition to Cas9 and gRNA. The incorporation of the gene of interest into the genome happens via a homologous-dependent repair mechanism. Most studies utilize plasmid-based endogenous expression of Cas9 and gRNA (Peng et al., 2014; Soares Medeiros et al., 2017). However, studies have reported the toxicity and instability due to the transgenic expression of Cas9 (Peng et al., 2014). Alternatively, the Cas9-gRNA ribonucleoprotein complex-based genome editing method was established in kinetoplastids (Beneke et al., 2017; Soares Medeiros et al., 2017; Verruto et al., 2018).

Here, we optimized electroporation-based transfection methodology to improve heterologous gene expression in *P. marinus*. Furthermore, using the optimized transfection protocol, we successfully delivered Cas9-gRNA ribonucleoprotein coupled with dDNA into the *P. marinus* wild-type trophozoites and tagged the *PmMOE1* gene with *GFP* at the C-terminus to achieve mutants phenotypically similar to previously reported *P. marinus* mutant strain (PRA-393) (Fernández Robledo et al., 2008).

## Results

### Plasmid Amount and Cell Number Optimization Experiments

**FIGURE 1:**
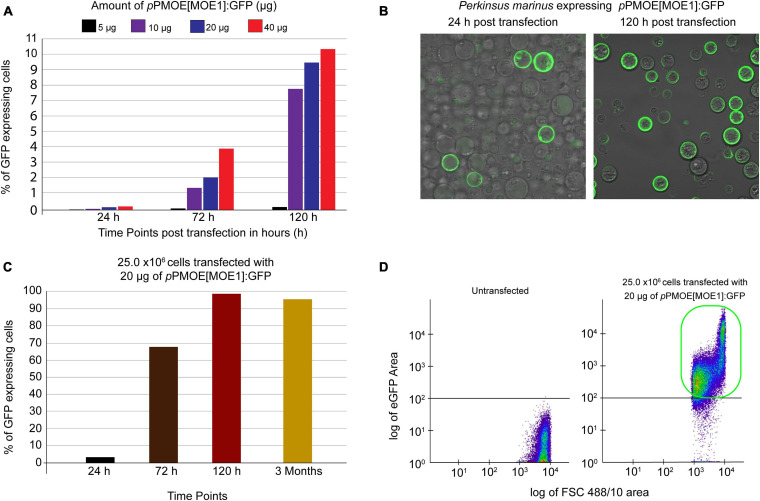
Plasmid amount and cell number optimization studies. **(A)** Fifty million parasites transfected with 5 μg (black bar), 10 μg (purple), 20 μg (blue), and 40 μg (orange) of *p*PmMOE[MOE1]:GFP, respectively. Bar graphs showing that the %GFP-positive cells (y-axis) were detected by flow cytometry at 24, 72, and 120 h post-transfection time points (x-axis). **(B)** Twenty-five million parasites transfected with 20 μg of *p*PmMOE[MOE1]:GFP 24 and 120 h post-transfections. **(C)** Twenty-five million parasites were transfected with 20 μg of *p*PmMOE[MOE1]:GFP, respectively. Bar graphs showing the %GFP-positive cells (y-axis) detected by flow cytometry at 24 (black bar), 72 (brown bar), 120 h (dark red bar), and 3 month (yellow bar) post-transfection time points (x-axis). **(D)** The scattered plot from FCM showing no GFP expression in untransfected controls and 98% GFP-positive cells in 25.0 × 10^6^ cells transfected with 20 μg of *p*PmMOE[MOE1]:GFP indicated in the green box.

To increase the heterologous gene expression efficiency, we used the previously developed Lonza-based electroporation method (Fernández Robledo et al., 2008). In the first round of optimization, we maintained 50.0 × 10^6^ *P. marinus* trophozoites per transfection. We optimized the *p*PmMOE[MOE1]:GFP plasmid amount with a twofold increase (5.0, 10.0, 20.0, and 40.0 μg). In all the cases, green fluorescent cells were observed under a UV-microscope as early as 24 h post-transfection. The flow cytometer was used to detect the number of GFP-positive cells. The trophozoites transfected with 5 and 10 μg of *p*PmMOE[MOE1]:GFP were detected as 0.002 and 0.03%, respectively. Parasites transfected with 5.0 μg of *p*PmMOE[MOE1]:GFP yielded 0.05 and 0.2% GFP-positive cells at 72 and 120 h time points, respectively (Figure 1A, black bar). Furthermore, parasites transfected with 10.0 μg of *p*PmMOE[MOE1]:GFP yielded 1 and 7.7% GFP-positive cells at 72 and 120 h time points, respectively (Figure 1A, purple bar). Interestingly, at 72 h post-transfection, parasites transfected with 40 μg (Figure 1A, orange bar) of *p*PmMOE[MOE1]:GFP yielded 2 × higher GFP-positive cells compared with parasites that received 20 μg (Figure 1A, blue bar). However, to our surprise, at 120 h post-transfection, we have detected 9 and 11% of GFP-positive cells in the cases of parasites transfected with 20 and 40 μg of the plasmid, respectively (Figure 1A, blue and orange bars). Observing the plateau of GFP-positive cells when parasites were transfected with 20 and 40 μg, we have decided to move on with the 20 μg of plasmid for cell number optimization.

In the second round of optimization, keeping the amount of *p*PmMOE[MOE1]:GFP plasmid constant at 20 μg/transfection, we varied *P. marinus* trophozoites cell number by a twofold increase between 1.56 × 10^6^ and 50.0 × 10^6^ cells/transfection. In this case, using the confocal microscope, we observed that 25.0 million parasites transfected with 20 μg of the plasmid yielded the highest levels of GFP-expressing cells qualitatively at 24- and 120 h post-transfection (Figure 1B). We took advantage of the flow cytometer and detected 2% of GFP-positive cells (Figure 1C, black bar) as early as 24 h and 68% in 72 h (Figure 1C, brown bar) and achieved 98% of GFP-positive cells 120 h post-transfection (Figure 1C, red bar). The trophozoites were monitored for 3 months, where we detected a constant 95% GFP-positive cells [Figure 1C, light brown bar and Figure 1D (highlighted in the green box)].

### Comparison of Proprietary and Non-proprietary Transfection Reagents and Materials

**FIGURE 2:**
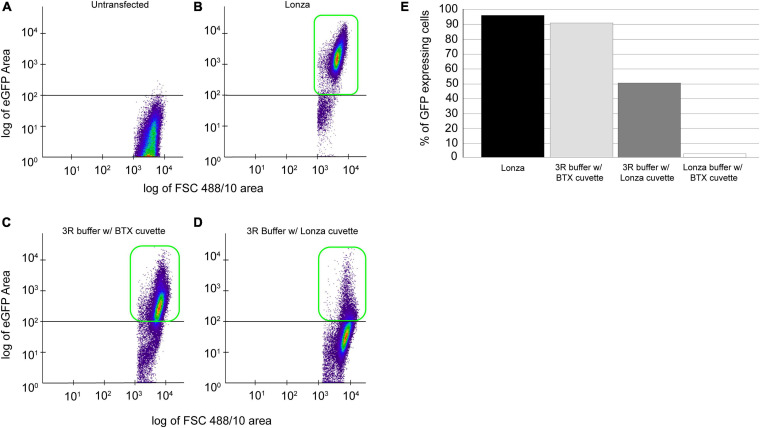
Comparison of proprietary and non-proprietary transfection. Cells, 25 × 10^6^ cells transfected with 20 μg of *p*PmMOE[MOE1]:GFP plasmid using proprietary and non-proprietary protocols. **(A)** Flow cytometry scattered plot of untransfected (wild-type) cells, no GFP expression detected. **(B)** The scattered plot of flow cytometry, identifying GFP-positive cells in transfection performed using the proprietary Lonza method. **(C)** Scatterplot representation of GFP-positive cells in transfection performed using 3R buffer and BTX cuvette. **(D)** Scatterplot showing the GFP-positive cells when transfected with 3R buffer utilizing Lonza cuvette. **(E)** Bar graph showing the % of GFP-positive cells when transfected with the Lonza system (black bar), 3R buffer in combination with BTX cuvette (gray bar), 3R buffer using Lonza cuvette (dark gray), and Lonza buffer with BTX cuvette (white bar).

To establish an affordable and reliable transfection methodology, we tested non-proprietary protocols, such as using 3R buffer with Lonza and commercial cuvettes (BTX Disposable Cuvettes Plus), and Lonza buffer-based transfection utilizing Lonza cuvette and commercial cuvette. In all the cases, 25 million cells were transfected with 20 μg of plasmid, and GFP-positive cells were detected 120 h post-transfection using flow cytometry (Figure 2A). As expected, we identified 98% of GFP-positive cells using a proprietary Lonza system (Figures 2B,E, black bar). To our surprise, flow cytometry evaluated 90% of GFP-positive cells using 3R-buffer and BTX cuvette (Figures 2C,E, light gray bar). By using a 3R buffer in combination with a Lonza cuvette, we detected 48% of GFP-positive cells (Figures 2D,E, dark gray bar). Finally, transfection utilizing Lonza–buffer and BTX cuvette yielded a meager 2% GFP-positive cells (Figure 2E, white bar).

### SpCas9-RNP and sgRNA Mediated GFP Knock-In in *Perkinsus marinus* Trophozoites

**FIGURE 3:**
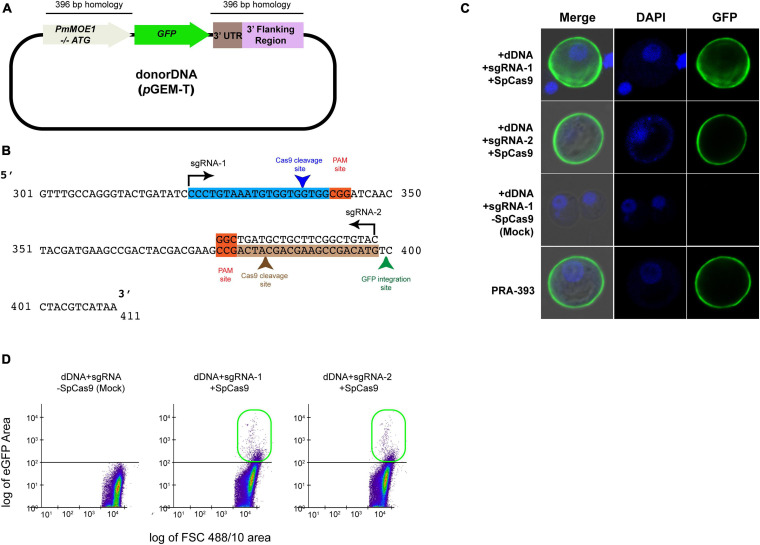
SpCas9-RNP and sgRNA-mediated GFP knock-in in *P. marinus* trophozoites. **(A)** Schematic representation of dDNA with 396 bp homology on the 5′ and 3′ of the GFP coding sequence. **(B)** Schematic representation of showing the guide RNA target sites on *PmMOE1* coding sequence sgRNA-1 targets the top strand indicated by the arrow direction; sgRNA-2 targets the bottom strand indicated by the arrow direction. **(C)** Confocal microscopy panel showing successful GFP expression in cells transfected with sgRNA-1/SpCas9 and sgRNA-2/SpCas9, showing localization pattern similar to the PRA-393 MOE-GFP mutant strain. **(D)** The scattered plot from FCM showing no GFP expression in mock (dDNA+sgRNA alone) control and 0.2% GFP-positive cells knocked in using sgRNA-1, and 0.35% in case of sgRNA-2 indicated in the green box.

To establish an HDR-based gene-editing method, we generated a dDNA plasmid containing 396 bp of *PmMOE*1 coding sequence lacking a start codon on the 5′ of the GFP coding sequence. Furthermore, there are 396 bp of 3′ UTR of *PmMOE1* at the 3′ of the GFP-coding sequence. The dDNA with GFP and templates was amplified using PCR from previously reported plasmid *p*PmMOE[MOE1]:GFP (schematic representation in Figure 3A; Fernández Robledo et al., 2008). The sgRNA targeting at position 314 on the top strand (sgRNA-1) and another sgRNA targeting position 395 on the bottom strand (sgRNA-2) of the *PmMOE1* coding sequence were designed using the Benchling software (Figure 3B). Twenty-five million *P. marinus* trophozoites were transfected with 20 μg of SpCas9 and sgRNA (1:1) along with 20 μg of dDNA. The parasites transfected with sgRNA-1/SpCas9 and sgRNA-2/SpCas9 and dDNA exhibited GFP expression 24 h post-transfection (Figure 3C, +sgRNA-1+dDNA+SpCas9 and +dDNA+sgRNA-2+SpCas9), showing a similar pattern of GFP mutant strain PRA-393 (Figure 3C, PRA-393 panel). The parasites transfected without SpCas9, only with dDNA and sgRNA used (i.e., +dDNA+sgRNA) as mock transfection, did not show GFP expression (Figure 3C, mock transfection panel). Two months post-transfection, using the flow cytometer, we detected 0.2% GFP-positive cells in the experiment transfected with sgRNA-1+dDNA+SpCas9. Furthermore, parasites transfected with sgRNA-2+dDNA+SpCas9 complex yielded 0.26%. GFP-positive cells were not detected in mock transfections (Figure 3D).

### Sorting *Perkinsus marinus* GFP-Positive Cells for Genotyping Validation

**FIGURE 4:**
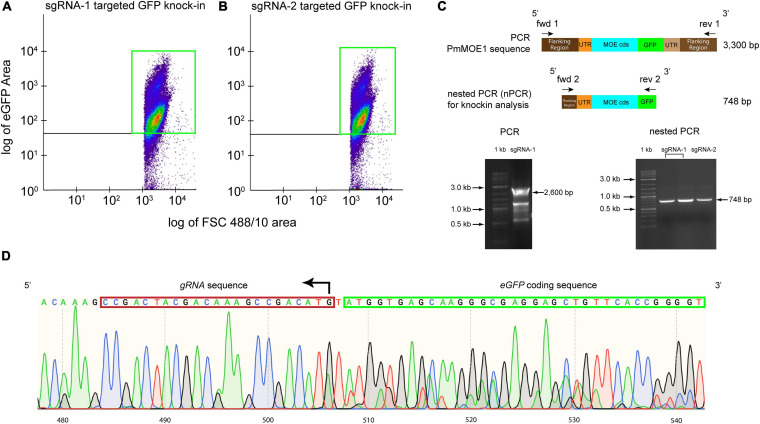
Sorting *P. marinus* GFP-positive cells for endogenous PmMOE1 C-terminus GFP tagging analysis. **(A)** Scattered plot showing 81% GFP-positive cells indicated with a green box in the experiment where cells transfected with sgRNA-1-Cas9. **(B)** Scattered plot showing 87% GFP-positive cells indicated with a green box in the experiment where cells transfected with sgRNA-1-Cas9. **(C)** The PCR intended to amplify the knock-in (expected sized 3,300 bp) using Fwd 1 and Rev 1 primers resulting in the amplification of the wildtype 2,600 bp amplicon (left panel). This PCR product was used as a template in the nested PCR (nPCR) to confirm the GFP knock-in using Fwd 2 and Rev 2 primers, which yielded the expected 748 bp amplicon (right panel), 1 kb Plus DNA Ladder (New England Biolabs, Ipswich, MA, United States). **(D)** Sequencing results of the nPCR product from the sgRNA-2 targeted GFP knock-in experiment.

Ten thousand GFP-positive cells from the sgRNA-1 and sgRNA-2 transfections were sorted and cultured for 3 months. Flow cytometry analysis detected approximately 81% of GFP-positive cells transfected with sgRNA-1 (Figure 4A) and 87% of GFP-positive cells transfected with sgRNA-2 (Figure 4B), respectively. Attempts of amplification of the knock-in (expected size 3,300 bp) resulted in around 2,600 bp amplicon, which would include the 5′ flanking, 5′ UTR, PmMOE1, but not the GFP knock-in (Figure 4C, 2,600 bp arrow and Supplementary Figure 2A) suggesting that the knock-in of GFP was less represented compared with the wild type. Consequently, we diluted the PCR product and run a nested PCR with specific primers targeting the putative knock-in; sequencing of the nested PCR product (748 bp) confirmed the accurate integration by HDR (Figure 4C, 748 bp arrow and Supplementary Figure 2B). The chromatogram validated the successful knock-in of GFP at the C-terminus *PmMOE1* (Figure 4D).

## Discussion

*Perkinsus marinus*, a marine protozoan parasite, causing devastating infection to eastern oysters, is currently under development as a model organism for the protozoan parasite of mollusks (Yadavalli et al., 2020). The availability of axenic culture and transfection methodology, the parasite’s ability to naturally trigger an immune response in mice, and phylogenetic affinities drove us to use it to express apicomplexan genes. However, these attempts were met with variable success (Wijayalath et al., 2014; Cold et al., 2017). The other laboratories often report the inconsistency of the gene expression.

The original transfection method uses 5 μg of plasmid and 50.0 × 10^6^ cells; we started by increasing the plasmid amount by 2-fold to 40 μg. The plasmid amount increased in higher GFP-positive cells, especially cells transfected with 20 and 40 μg of the plasmid. In all the cases, fluorescent cells were observed as early as 24 h post-transfection. Cells need 3 days to recover and for the GFP expression to be quantifiable. The cells transfected with 40 μg yielded twofold higher GFP-positive cells than cells transfected with 20 μg of plasmid after 72 h. However, at 120 h, the number of GFP-expressing cells plateaued to 10% in both cases, indicating that the number of cells also affects transfection efficiency. We determined that 25.0 × 10^6^ cells transfected with 20 μg of plasmid resulted in 98% GFP-positive cells 120 h post-transfection. The Nucleofector^TM^ 2b uses cuvettes, and the transfection occurs in a 100 μl reaction, and it appears that the delivery of the electrical pulse is optimal when 25.0 × 10^6^ cells are used.

Interestingly, cell numbers above and below 25.0 × 10^6^ cells resulted in quite a low transfection efficiency (Supplementary Figure 1). We also could transfect *P. marinus* cells with the non-proprietary transfection buffer (3R buffer), which provides an efficiency above 40% and provides savings when the research budgets are tight. Experiments utilizing the combination of non-proprietary transfection buffer and BTX cuvettes were successful, although with a low number of transfectants. We observed more than 90% of GFP-expressing cells even at 3 months post-transfection in all the cases, suggesting a stable GFP expression.

The CRISPR/Cas9 methodology is broadly adopted by numerous parasitology labs around the world (Mali et al., 2013; Ghorbal et al., 2014; Peng et al., 2014; Shen et al., 2014; Sollelis et al., 2015; Janssen et al., 2018; Lin et al., 2019). Utilizing the optimized conditions, we took a step further to develop the CRISPR/Cas9-based gene editing methodology for *P. marinus*. For the proof of concept, we targeted the *PmMOE1* gene that has a defined phenotype when tagged with GFP (Fernández Robledo et al., 2008). We were able to detect fluorescent trophozoites within 18 h of delivering the CRISPR/Cas9 system components. Lack of GFP expression in the dDNA alone transfection (lacking CRISPR/Cas9 components) rules out the possibilities of non-homologous recombination in frame with any expressed gene; however, with this fluorescence screening, plasmid fragmentation and integration at the transposable element sites cannot be excluded. The GFP expression pattern in the transfectants was similar to that of *P. marinus* PRA393 (Figure 3C). GFP-positive cells sorted from sgRNA-1/Cas9 and sgRNA-2/Cas9 experiments were PCR amplified to check for the knock-in of the GFP. Interestingly, the PCR in the sorted cells did not result in the 3,300 bp amplicon. However, the nested PCR produced the expected size amplicons whose direct sequences confirmed the successful knock-in of GFP at the C-terminus of *PmMOE1*.

In the protozoan parasites with a large trajectory of genetic manipulation, the trend is to build a plasmid vector that incorporates both the expression of Cas9 and the sgRNA or even generate a mutant conditionally expressing *Cas9.* We chose to deliver the CRISPR/Cas9 components, including the SpCas9 nuclease, directly by electroporation. The data reported here are from a single trial targeting *PmMOE1* using 25.0 × 10^6^ log-phase trophozoites, 20 μg of dDNA, 10 μg of sgRNAs, and SaCas9 nuclease chosen based on Beneke et al. (2017) and Soares Medeiros et al. (2017) resulted in a successful knock-in. The optimization was outside of the scope of this study; more robust optimization focusing on the amount of guide RNA and utilization of single-stranded linear vs. double-stranded dDNA, would likely result in an optimized protocol (Beneke et al., 2017; Markus et al., 2019). With this CRISPR/Cas9 system and several *Perkinsus* spp. genomes being available (Bogema et al., 2020), we now have the tools to interrogate these genomes and improve the experimental design of sgRNA to target additional genes.

Genome editing tools like CRISPR/Cas9 in parasite biology is used for gene disruption, fluorescent tagging, and single nucleotide mutation incorporation to study genes involved in the parasite growth, invasion, and drug resistance (Wagner et al., 2014; Di Cristina et al., 2017). For example, in *Plasmodium falciparum* study development of artemisinin-resistant parasite by single-nucleotide substitution, identification of the multidrug resistance mutation 1 (*PfMDR1*) in response to the drug ACT-451840 and incorporation of a point mutation in the *PfATP4* gene to generate the drug-resistant strain were all possible by utilizing the CRISPR/Cas9 system (Ghorbal et al., 2014; Ng et al., 2016; Crawford et al., 2017). In *Toxoplasma gondii*, CRISPR/Cas9 is widely used in high-throughput and genome screening studies to identify essential genes involved in parasite invasion and antiparasitic drug candidates (Di Cristina et al., 2017; Di Cristina and Carruthers, 2018). CRISPR/Cas9-based knock-out studies in *Cryptosporidium parvum* are used to understand the mechanism of the parasite’s resistance to antifolate drugs and nutrient acquisition pathways (Vinayak et al., 2015; Pawlowic et al., 2017, 2019).

*P. marinus* genome encodes for 23,454 genes embedded in 17,000 supercontigs. However, tetra-polyploidy pose a significant bottleneck for the assembly (El-Sayed et al., 2007; Bogema et al., 2020). Proteome studies identified that *P. marinus* possess 4,073 non-redundant hypothetical proteins, of which 36 and 27% are involved in metabolic and cellular processes, respectively (Marcia et al., 2017). Additionally, the rhoptry proteins such as serine–threonine kinases, protein phosphatases, proteosomes, and a virulent candidate merozoite surface protein 3, which are known to play a crucial role in parasite invasion and cell–cell communication during the invasion in *P. falciparum* were also identified in *P. marinus*. Studies so far reported that *P. marinus* possess extracellular proteins such as high molecular weight cell wall protein 1 (Montes et al., 2002); glycosylation, mucin, and sugar-binding domain protein Pmar_XP_002783417.1 encoded by *Pmar_PMAR006943*; sensory signal transduction-related histidine kinase encoded by *Pmar_PMAR009211*; and a family of cysteine-rich modular proteins whose function in the parasite life cycle are yet to be investigated (Montes et al., 2002). Furthermore, apoptotic genes such as apoptosis inhibitory molecule (Fas), apoptosis-inducing factor (Tadesse et al., 2017; Lau et al., 2018b), peroxiredoxin, and superoxide dismutase are shown to favor parasite survival by reducing the host cell (Schott and Vasta, 2003; Schott et al., 2003; Box et al., 2020). The function of these apoptotic genes responsible for the disease in the oysters is limited.

The CRISPR/Cas9 method developed here can be used to understand the localization and protein–protein interactions in the parasite’s life cycle and generate the transgenic parasite model. The natural adjuvant ability of the *P. marinus* and its potential as a novel oral vaccine platform, efforts for the expression of heterologous antigens, always relied on one plasmid *p*[MOE]:EK-His-GFP (Cold et al., 2017), with potential for a monovalent vaccine expression. With the availability of fast and robust CRISPR/Cas9, we can now express multiple heterologous genes and develop *P. marinus* as a polyvalent oral vaccine delivery system. Considering the precision and efficiency of CRISPR/Cas9 in gene editing, this opens doors for discovering new treatments and therapeutic discoveries. The system can also be used in clinical and population validation studies to identify new antiparasitic agents (Visscher et al., 2017). Finally, the CRISPR/Cas9 system can be applied to generate non-pathogenic and immunogenic parasites for the immunization and vaccination studies (Hollingdale and Sedegah, 2017).

## Materials and Methods

### *Perkinsus marinus* Cell Culture

Experiments were carried out with cultures of the wild-type *P. marinus* CB5D4 (ATCC#PRA-240) (Shridhar et al., 2013) maintained in DME:Ham’s F12 (1:2) supplemented with 5% fetal bovine serum (FBS), in a 25 cm^2^ (5–8 ml) polystyrene canted neck cell culture flasks with vent caps (Corning^®^, Corning, New York, United States) at 24–28°C in a microbiology incubator as reported elsewhere (Gauthier and Vasta, 1995). Trophozoites in the log phase (OD_595_ = 0.4–0.5) were aliquoted in Eppendorf tubes to contain 1.56 × 10^6^, 3.13 × 10^6^, 6.25 × 10^6^, 12.50 × 10^6^, 25.0 × 10^6^, and 50.0 × 10^6^.

### *Perkinsus marinus* Transfection

The transfection vector *p*PmMOE[MOE1]:GFP (former *p*PmMOE-GFP) (Fernández Robledo et al., 2008) was propagated in *Escherichia coli* JM109. Plasmid minipreps were prepared using a commercial kit (E.Z.N.A.^®^ Plasmid mini Kit I, Omega-Tek, Norcross, GA, United States), and DNA concentration and purity were estimated with a Nanodrop^TM^ 1000 spectrophotometer (Thermo Fisher Scientific, Waltham, MA, United States). The isolated plasmid DNA was air dried using speedVac for all the experiments. *P. marinus* cells were prepared following the Cell Line Optimization Nucleofector Kit before electroporation using the Nucleofector^TM^ 2b (Lonza, Walkersville, MD, United States). For all the experiments, we used the pre-set program D-023 and Lonza’s solution V (Fernández Robledo et al., 2008). Briefly, dried plasmid was resuspended in 100 μl of Solutio V containing supplement 1. We tested 5, 10, 20, and 40 μg of *p*PmMOE[MOE1]:GFP with 50 million *P. marinus* cells. Once the optimal plasmid amount was established (20 μg), we tested it with variable *P. marinus* cell number (1.56 × 10^6^, 3.13 × 10^6^, 6.25 × 10^6^, 12.5 × 10^6^, 25.0 × 10^6^, and 50.0 × 10^6^). Immediately after electroporation, the individual electroporation cuvettes’ contents were transferred to a 24-well plate, each well containing 1 ml of DME:Ham’s F12 (1:2) supplemented with 5% FBS (Gauthier and Vasta, 1995). The cuvettes were gently washed with 500 μl of fresh culture medium and pooled with those wells corresponding to each original sample. We also tested the non-proprietary transfection buffer (3R buffer)-based transfection protocol (Faktorová et al., 2020). The 3R-transfection buffer, composed of 200 mM Na_2_HPO_4_, 70 mM NaH_2_PO_4_, 15 mM KCl, and 150 mM HEPES, was prepared and pHed to 7.3. Dried 20 μg of circular *p*PmMOE[MOE1]:GFP plasmid was resuspended in 60 μl of milliQ water. Once dissolved, 35 μl of 3R transfection buffer and 10 μl of 1.5 mM CaCl_2_ were added (Protocols.io). Twenty-five million *P. marinus* trophozoites were transfected.

### Protospacer Adjacent Motif-Target Site Selection and Donor DNA Construction

PAM-target site selection was identified using the PmMOE1 sequence (*Pmar_PMAR027036*) and the software (Benchling, Inc.)^1^. The output sequences were searched using BLASTx (NCBI-Blast, 2021) against the *P. marinus* nr database (RefSeq assembly: GCF_000006405.1), which predicted *Pmar_PMAR025337* as another possible target. Based on the string searches, the PAM sites were rated according to their“uniqueness.” Single-guide RNA (sgRNA) targeting positive strand at position 339 of PmMOE1 CDS (sgRNA-1) 5′-CCC TGT AAA TGT GGT GGT GG-3′ and sgRNA targeting negative strand at position 382 of PmMOE CDS (sgRNA-2) 5′-CAT GTC GGC TTC GTC GTA GT CGG-3′ with unique PAM sequence “CGG” were synthesized (Synthego, Silicon Valley, CA, *United States*). Although the sgRNA-2 sequence identified three target sites on PmMOE1 CDS, the sequence reported here is the only fragment that exhibited 100% complementarity. The dDNA was amplified from *p*PmMOE[MOE1]:GFP (Fernández Robledo et al., 2008) using primers forward 5′-CGC TTC ATT GTT GGT CTG TAC–3′ and reverse 5′-CAG TAC GAA ATT ACG CGA GAT G–3′. The amplicon was cloned into the *p*GEM^®^-T vector by T-A cloning (*p*GEM-T Vector Systems, Promega Corporation, Madison, WI), propagated in *Escherichia coli* JM109 (L1001, Promega), and sent for sequencing. Plasmid minipreps were prepared using a commercial kit (E.Z.N.A.^®^ Plasmid Mini Kit I, Omega Bio-Tek, Norcross, GA, *United States*), and DNA concentration and purity were estimated with a Nanodrop 1000 spectrophotometer.

### RNP Complex and Donor DNA Delivery Into *Perkinsus marinus*

*Perkinsus marinus* cells were prepared following the Cell Line Optimization Nucleofector Kit protocol before electroporation. Using the Nucleofector^TM^ 2b, 10 μg of *Streptococcus pyrogenes* Cas9 (SpCas9) nuclease TrueCut^TM^ Cas9 protein v2 (Thermo Fisher Scientific, Vilnius, Lithuania) and 10 μg of sgRNAs (Synthego, Silicon Valley, CA, United States) were mixed in 100 μl of Lonza’s solution V and incubated at room temperature for 15 min for hybridization of sgRNA and Cas9 protein (Beneke et al., 2017). Twenty micrograms of dried dDNA plasmid were resuspended with a SpCas9–gRNA complex and electroporated into 25.0 × 10^6^ *P. marinus* trophozoites, using pre-set D-023 program (Fernández Robledo et al., 2008). Immediately after electroporation, the individual electroporation cuvettes’ contents were transferred to a 12-well plate, each well containing 1 ml of DME:Ham’s F12 (1:2) supplemented with 5% FBS, to allow cells to recover (Gauthier and Vasta, 1995). Upon identifying the fluorescent cells, the cells were spun down at 1,000 × *g* for 5 min at room temperature and resuspended into fresh media. Cells were screened for green fluorescence at 24, 72, and 120 h, and 2, 4, and 6 weeks post-transfection using confocal microscopy and flow cytometry.

### DNA Isolation and Genotyping for GFP Knock-In by DNA Sequencing

Upon observing green fluorescent *P. marinus* trophozoites, cells were allowed to recover for 1 week. The genomic DNA from *P. marinus*:wild-type (PRA-240), GFP-mutant (PRA-393), parasites transfected with dDNA alone, lacking CRISPR elements (sgRNA and Cas9), and parasites transfected with sgRNA-1 and sgRNA-2 with Cas9 were isolated using E.Z.N.A.^®^ tissue DNA kit (Norcross, GA, United States) according to the manufacturer protocol. The purity and concentration of isolated DNA were analyzed using Nanodrop^TM^ 1000 spectrophotometer. For DNA genotyping, the primer pair Fwd 1 5′-CTC GTA ATG AGC CCA ACC AT–3′ and Rev 1 5′-GGA GGA CTT GAG GCT CTG TG 3′ (Fernández Robledo et al., 2008) were designed using the available supercontig (Ensembl, 2021) results in 2,600 bp of *PmMOE1* (wildtype) and would yield 3,300 bp after successful GFP knock-in. To identify the GFP knock-in site at the 3′ PmMOE CDS, we designed primers spanning 136 bp of the 5′ flanking region, PmMOE1 CDS, and 201 bp of the GFP sequence (Fwd 2 5′-TGT TGT AAG GCG AGA CGC TA–3′ and Rev 2 5′-GTA GGT CAG GGT GGT CAC GA–3′), respectively. Briefly, 50 ng of the gDNA and primers mentioned above were used to amplify by polymerase chain reaction. The amplicons were purified from the 1% agarose gel using the Zymoclean^TM^ Gel DNA Recovery kit (Tustin, CA, United States).

### Confocal Microscopy

Parasites were fixed with 3% paraformaldehyde (Thermo Fisher Scientific, preserved 37% reagent) for 15 min at room temperature. Parasites were washed three times at 1,000 × *g* for 5 min using 1 × phosphate-buffered saline (1 × PBS). Following the washes, parasites were treated with 0.1% Triton X-100 for 15 min and washed three times with 1 × PBS. The cells were stained with 25 μg/ml concentration of 4′,6-diamidino-2-phenylindole (DAPI) (Vector Laboratories, Burlingame, CA, United States). Excess DAPI was washed with 1 × PBS, and parasites were resuspended in the fresh 1 × PBS and placed in NunC^®^ Lab-Tek^®^ II (Millipore Sigma, Darmstadt, Germany) for live-cell imaging. Parasites were imaged at a total magnification of 630 × on Carl Zeiss LSM-700 multiphoton scanning laser microscope.

### Flow Cytometry Sorting and Analysis

The flow cytometry experiments were performed on the live parasite, using ZE5 Cell Analyzer; data was collected using Everest software version 2.0 and analyzed. A minimum of 100,000 events was collected for parasites based on forward and side scatterplot, and a singlet gate was applied to collect a minimum of 30,000 cells. BD Sciences Influx Cell Sorter (BD Sciences, NJ, United States) was used for cell sorting, and cells were sorted based on eGFP-positive gates.

## Data Availability Statement

The raw data supporting the conclusions of this article will be made available by the authors, without undue reservation.

## Author Contributions

RY and JF: conceptualization, methodology, writing—original draft preparation, and supervision. JF: validation, project administration, and funding acquisition. RY, KU, HW, AT, AS, DH, and JF: formal analysis, investigation, writing—review and editing, and visualization. RY and KU: resources. RY: data curation. All authors contributed to the article and approved the submitted version.

## Conflict of Interest

The authors declare that the research was conducted in the absence of any commercial or financial relationships that could be construed as a potential conflict of interest.

## Footnotes

1 https://www.benchling.com/

## Supplementary Material

Supplementary Figure 1 Cell number optimization studies.

Supplementary Figure 2 PCR and nested PCR based genotyping of *PMAR_Pmar027036* for the GFP knock in analysis. The genomic DNA was isolated from two different clones labeled as 4 and 5, which were hand-picked 3 months after the cell sorting from the experiments involved in the utilization of SgRNA-1 and SgRNA-2, respectively. **(A)** The PCR product showing the successful amplification of 2,600 bp of DNA sequencing containing flanking regions, MOE1 CDS encoded by *PMAR_Pmar027036* and the GFP. PCR product was diluted 100 times and used in the nested PCR to identify the knock in of the GFP, which successfully amplified the 748 bp of DNA fragment. The fragment was sequenced to confirm the successful knock in of GFP. **(B)** Repeat of the gel analysis of the nested PCR products from **(A)**, showing the DNA size of 748 bp.
