# Supplementary figures and images for: CRISPR/Cas9 Ribonucleoprotein-Based Genome Editing Methodology in the Marine Protozoan Parasite Perkinsus marinus
Source: Front Bioeng Biotechnol. 2021 Apr 9;9:623278. doi: 10.3389/fbioe.2021.623278 (PMC8062965; doi:10.3389/fbioe.2021.623278)

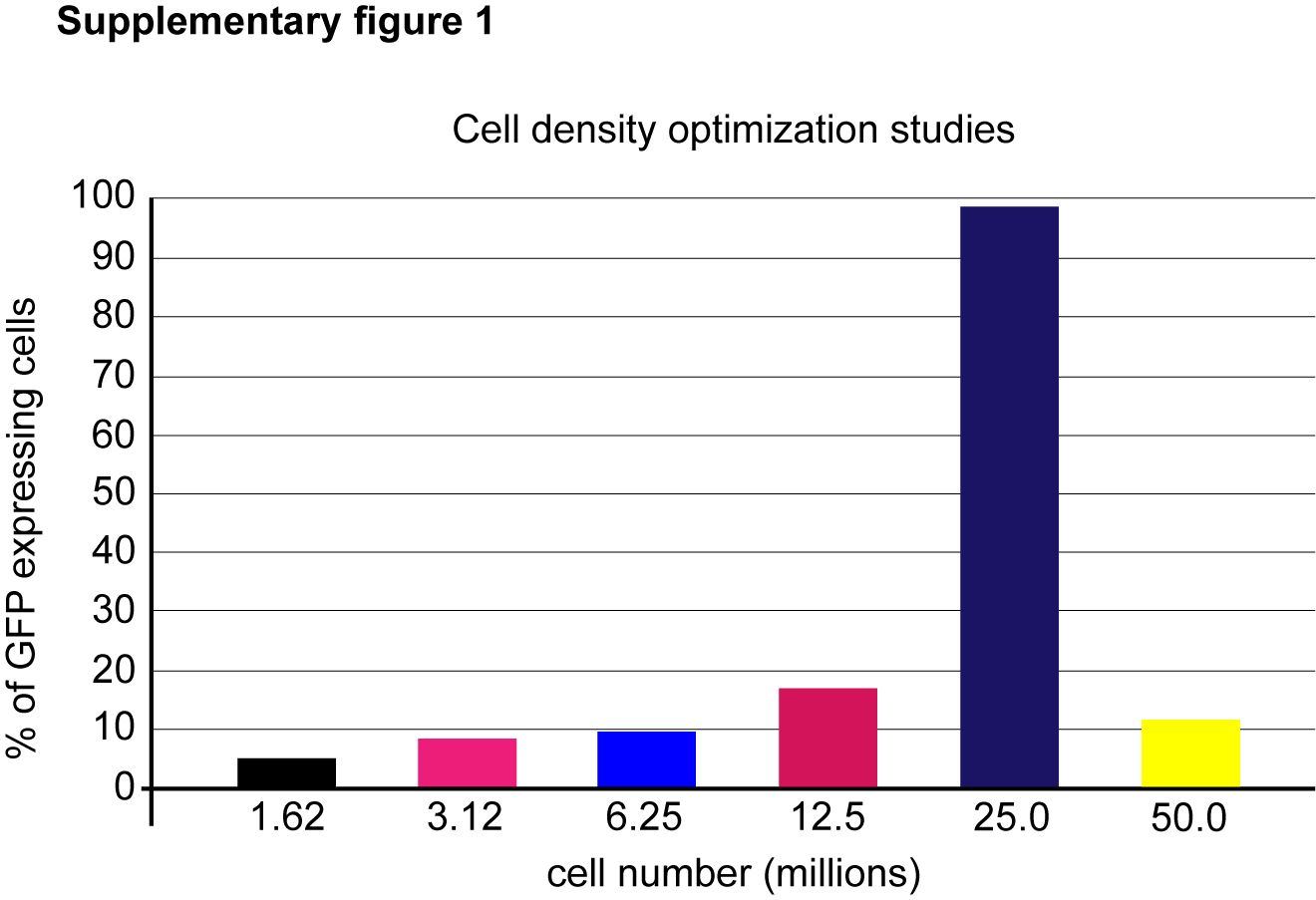

Supplement: Supplementary Figure 1 — Cell number optimization studies. [file Image_1.TIF]

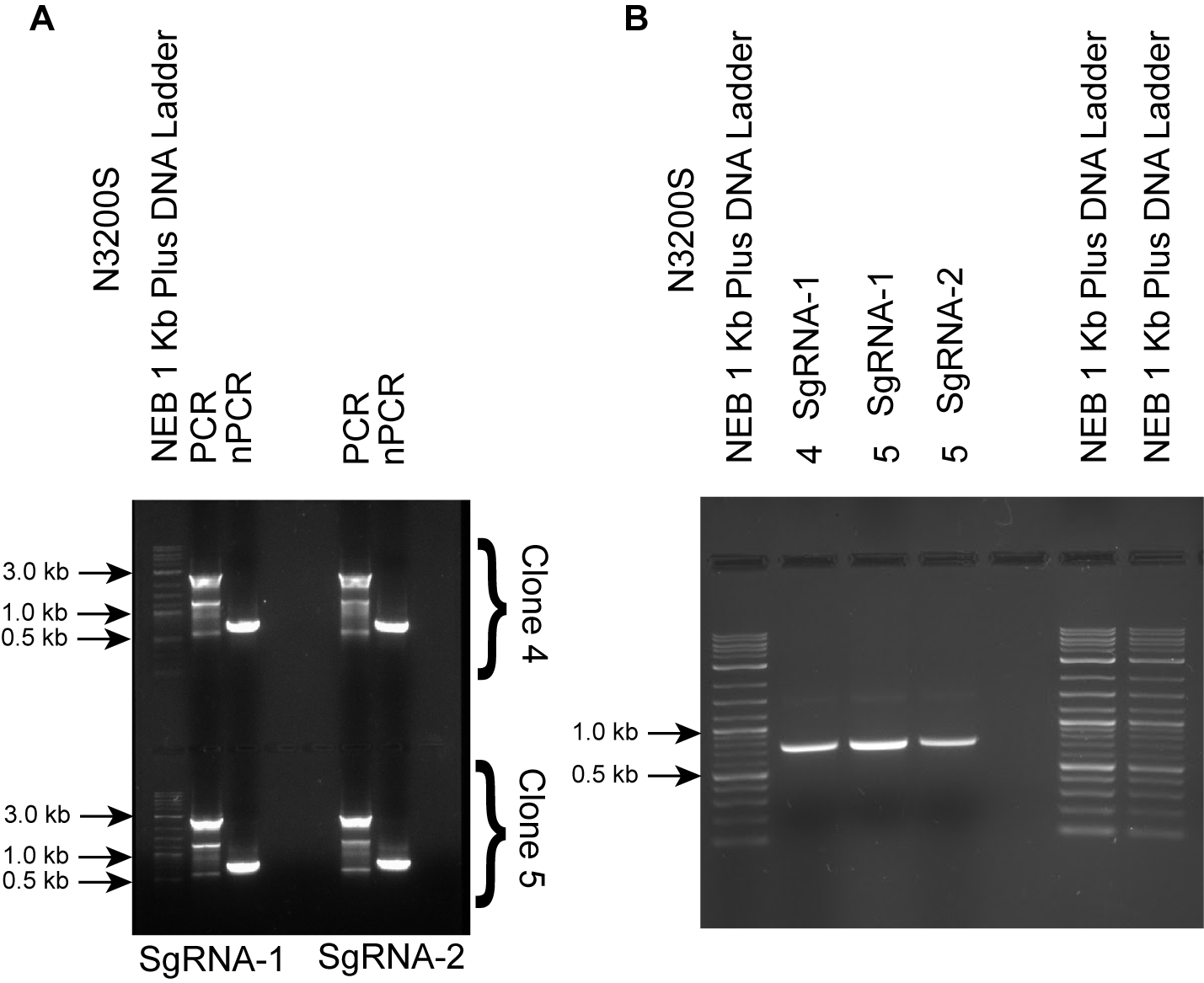

Supplement: Supplementary Figure 2 — PCR and nested PCR based genotyping of PMAR_Pmar027036 for the GFP knock in analysis. The genomic DNA was isolated from two different clones labeled as 4 and 5, which were hand-picked 3 months after the cell sorting from the experiments involved in the utilization of SgRNA-1 and SgRNA-2, respectively. (A) The PCR product showing the successful amplification of 2,600 bp of DNA sequencing containing flanking regions, MOE1 CDS encoded by PMAR_Pmar027036 and the GFP. PCR product was diluted 100 times and used in the nested PCR to identify the knock in of the GFP, which successfully amplified the 748 bp of DNA fragment. The fragment was sequenced to confirm the successful knock in of GFP. (B) Repeat of the gel analysis of the nested PCR products from (A), showing the DNA size of 748 bp. [file Image_2.TIF]
